# Genomic epidemiology and evolutionary analysis during XBB.1.16-predominant periods of SARS-CoV-2 omicron variant in Bangkok, Thailand: December 2022–August 2023

**DOI:** 10.1038/s41598-023-50856-0

**Published:** 2024-01-05

**Authors:** Jiratchaya Puenpa, Jira Chansaenroj, Kamol Suwannakarn, Yong Poovorawan

**Affiliations:** 1 Center of Excellence in Clinical Virology, Department of Pediatrics, Faculty of Medicine, Chulalongkorn University, Bangkok, Thailand; 2 Department of Microbiology, Faculty of Medicine, Siriraj Hospital, Mahidol University, Bangkok, Thailand; 3 FRS(T), The Royal Society of Thailand, Sanam Sueapa, Dusit, Bangkok, Thailand

**Keywords:** Microbiology, Virology, SARS-CoV-2, Sequencing, Phylogenetics

## Abstract

The growing occurrence of novel recombinants, such as XBB.1.16, has emerged and become predominant, raising concerns about the impact of genomic recombination on the evolution of severe acute respiratory syndrome coronavirus 2 (SARS-CoV-2). This study investigated the molecular epidemiological trends and evolution of the Omicron XBB.1.16 epidemic in Bangkok between December 2022 and August 2023. Partial spike and complete genome sequencing of SARS-CoV-2 samples collected from collaborating hospitals were performed. The analysis of 491 partial spike sequences identified 15 distinct lineages, with XBB.1.16 dominating the lineages beginning in March 2023. Phylogenetic analysis revealed at least four distinct XBB.1.16 lineages, suggesting multiple independent introductions into Bangkok. The estimated emergence of XBB.1.16 occurred approximately in January 2022, with an evolutionary rate of 0.79 × 10^–3^ substitutions per site per year. Monitoring the genomic epidemiology and evolution of XBB.1.16 is vital for the early detection of new strains or emerging variants, which may guide vaccine design and the inclusion of new vaccine strains.

## Introduction

Since its first emergence at the Huanan Seafood Wholesale Market in December 2019 in Wuhan, China, severe acute respiratory syndrome coronavirus 2 (SARS-CoV-2) has caused over 6 million confirmed deaths in over 3 years^1,2^. In early 2022, there was a notable surge in the global incidence of COVID-19 cases, marking a peak in case numbers^3,4^. In January 2022, over 63.5 million confirmed cases have been reported globally^3–6^. Subsequently, from the end of January to early March 2022, a consistent decline in new COVID-19 cases was reported^5,7^. As of 2023, the current patterns in globally reported COVID-19 cases suggest an underestimation of the actual number of global infections and reinfections^8,9^. In January 2023, there were nearly 20 million confirmed cases, followed by a decline to 4.8 million in February and a further decrease to 3.6 million patients in March^10–12^. From June to August, the reported number of COVID-19 cases remained consistently at one million each month^13–15^.

In Thailand, during early 2022, the number of reported COVID-19 patients with the omicron variant surged significantly, but it has continuously decreased since July 2022^16^. After the Songkran Festival (also known as the Thai New Year Festival) in April 2023, there was a tenfold increase in COVID-19 cases, creating potential opportunities for heightened transmission. The surge in cases was likely exacerbated by many individuals traveling back to their hometowns to partake in the festive celebrations^17–19^.

In the early stages of the pandemic, one of the initial SARS-CoV-2 mutations that rapidly spread worldwide was the D614G mutation (PANGO lineage B.1) in the spike (S) gene, which was first identified in Europe in January 2020^20^. As of now, the World Health Organization, along with national public health agencies, has designated five SARS-CoV-2 variants as variants of concern (VOCs) due to their significantly altered transmissibility or immune escape capabilities: Alpha (lineage B.1.1.7), Beta (B.1.351), Gamma (P.1), Delta (B.1.617.), and Omicron (B.1.1.529). The Delta and Omicron variants have rapidly spread worldwide, whereas Alpha, Beta, and Gamma have asserted dominance in specific regions. With mutations in both the receptor-binding domain (RBD) and the N-terminal domain (NTD), all these VOCs share a common N501Y mutation situated on the RBD, except for the Delta variant. This specific mutation increases the spike protein's affinity to angiotensin-converting enzyme 2 (ACE2) receptors, thereby strengthening the high infectivity^21^. The L452R mutation within Delta variants enhances spike stability, viral infectivity and fusogenicity, ultimately promoting viral replication^22^. With over 60 mutations, the Omicron variant possesses 15 mutations within the RBD^23^**.** The mutations in the NTD region, such as T76I, L141F, G142Y, 156–167 deletion, and R158G, have been observed to impact antibody binding efficiency, contributing to immune evasion^24^. The development of vaccines against SARS-CoV-2 represents a significant milestone in mitigating the effects of COVID-19. Nevertheless, the pandemic continues to persist, facilitating the accumulation of mutations throughout the entire SARS-CoV-2 genome due to ongoing viral transmission.

The XBB.1.16 subvariant has accounted for the substantial upsurge in COVID-19 cases in India in January 2023^25^. Its global prevalence has been steadily rising, marked by weekly increases, and the WHO has identified XBB.1.16 in at least 33 countries^26^. As a result of this surge in cases, the WHO officially categorized XBB.1.16 as a variant under monitoring status on 22 March 2023, following its initial designation as a variant of interest on 17 April 2023^27^. Nonetheless, there is currently no evidence indicating that it causes more severe illness.

In Thailand, six lineages were identified in the first quarter of 2020, including A, A.6, B, B.1, B.1.8, and B.58^28^. The B.1.36.16 variant was responsible for the predominant outbreak during the second wave (December 2020–January 2021), while the Alpha variant led the third wave (April–June 2021), Delta characterized the fourth wave (July–December 2021), and Omicron emerged during the fifth wave (January–March 2022)^29^. In July 2023, the new SARS-CoV-2 variant BA.2.86 was identified in the community through wastewater surveillance in Bangkok^30^. Since the emergence of the XBB.1.16 variant in March, the Ministry of Public Health (MoPH) has recorded a cumulative total of 24,292 confirmed cases^16–19^. During the period dominated by XBB.1.16, the number of infections was less than half of the outbreak attributed to the BA.2.75 variant in late 2022 (Figure S1).

The emergence and dominance of novel recombinants, like XBB.1.16, have raised concerns regarding the influence of genomic recombination on the evolution of SARS-CoV-2. This study investigated the molecular epidemiological trends and evolution of the omicron XBB.1.16 epidemic in Bangkok, Thailand, between December 2022 and August 2023.

## Results

### Molecular detection of SARS-CoV-2 lineages and sub-lineages

During the study period, we collected 491 RNA-positive SARS-CoV-2 samples from Bangkok residents, submitted to collaborating hospitals in Bangkok. These samples were amenable to partial spike sequencing, with an additional 50 samples chosen randomly for complete genome sequencing. Of the collected samples, 278 (57%) were from female patients and 213 (43%) were from male patients. Ten percent of the patients were aged 2 months to 10 years, 2% were aged 11–20 years, 16% were aged 21–30 years, 22% were aged 31–40 years, 14% were aged 41–50 years, 13% were aged 51–60 years, and 23% were older than 60 years (Table 1).

**Table 1:** Characteristics of SARS-CoV2 infected patients from December 2022 to August 2023.

	Bangkok (*n* = 491)	
	Number of cases	Percentage
Gender		
Male	213	43%
Female	278	57%
Age		
2mo-10 yr	50	10%
11-20 yr	10	2%
21-30 yr	79	16%
31-40 yr	108	22%
41-50 yr	67	14%
51-60 yr	63	13%
More than 60 yr	114	23%

**Figure 1:**
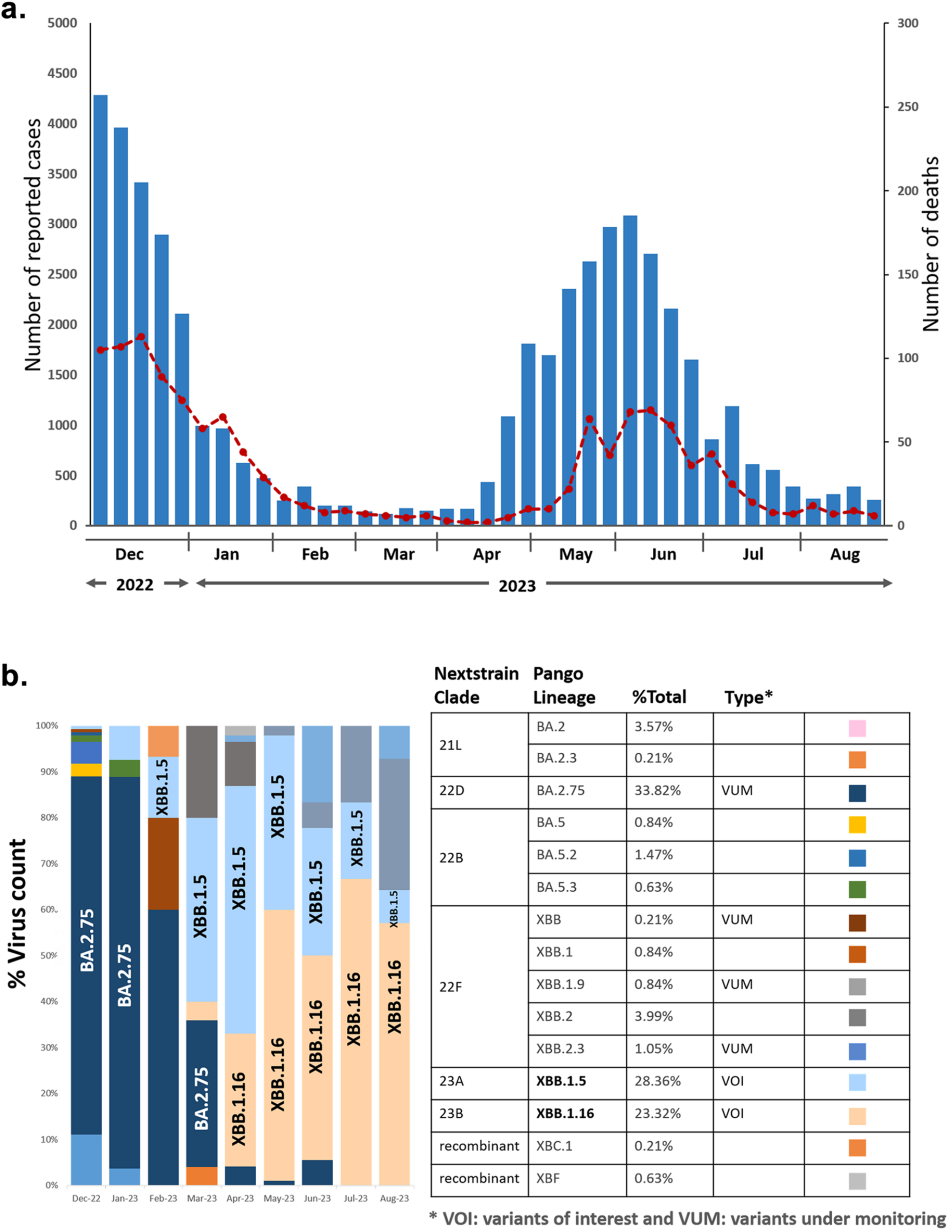
(**a**) Cases of confirmed SARS-CoV-2 infection reported in Thailand^31^. (**b**) Timecourse of omicron variant distribution in Bangkok, Thailand, between December 2022 and August 2023.

The Ministry of Public Health Thailand (MoPH) report covering the SARS-CoV-2 outbreak from December 2022 to August 2023 includes 49,139 confirmed cases and 1,279 deaths. (Fig. 1a)^31^. In 2023, a large increase in COVID-19 outbreaks was observed during the summer period, reaching a peak between May and June. Reports of COVID-19 cases in Thailand from February to early April were consistently low, with weekly numbers below 500 cases. The number of deaths from the COVID-19 outbreak in July 2023 declined by half compared to December 2022.

A comprehensive analysis of the 491 SARS-CoV-2 partial spike sequences generated identified 15 distinct lineages (Fig. 1b). Among these, 33.82% were identified as BA.2.75, 28.36% as XBB.1.5, 3.99% as XBB.2, 3.57% as BA.2, 1.47% as BA.5.2, and 1.05% as XBB.2.3. Five variants showed frequencies below 1%, including BA.5 (0.84%), XBB.1 (0.84%), XBB.1.9 (0.84%), BA.5.3 (0.63%), and BA.2.3 (0.21%). Additional lineages, specifically XBC.1 (0.21%) and XBF (0.63%) were also detected in February 2023 and April 2023 respectively.

During the study period, 23.32% of patients presented with XBB.1.16 infection. A significant increase in XBB.1.16 variants have been detected in the current study, with 4% in March, 29.0% in April, 58.9% in May, and peaking at 66.7% in July 2023. XBB.1.16 persisted as the predominant variant identified in the outbreak throughout August.

Figure S2 shows the evolutionary relationships among 491 Thai SARS-CoV-2 partial spike sequences and the corresponding emergence dates in Thailand. The Bayesian time-scaled phylogenetic analysis calculated an average evolutionary rate across the genome of 4.04 × 10^−3^ nucleotide substitutions per site per year (sub/site/year), with a 95% highest density interval (HDI) ranging from 2.75 × 10^−3^ to 5.40 × 10^−3^.

### Time-scaled phylogenetic reconstruction of SARS-CoV-2

**Figure 2:**
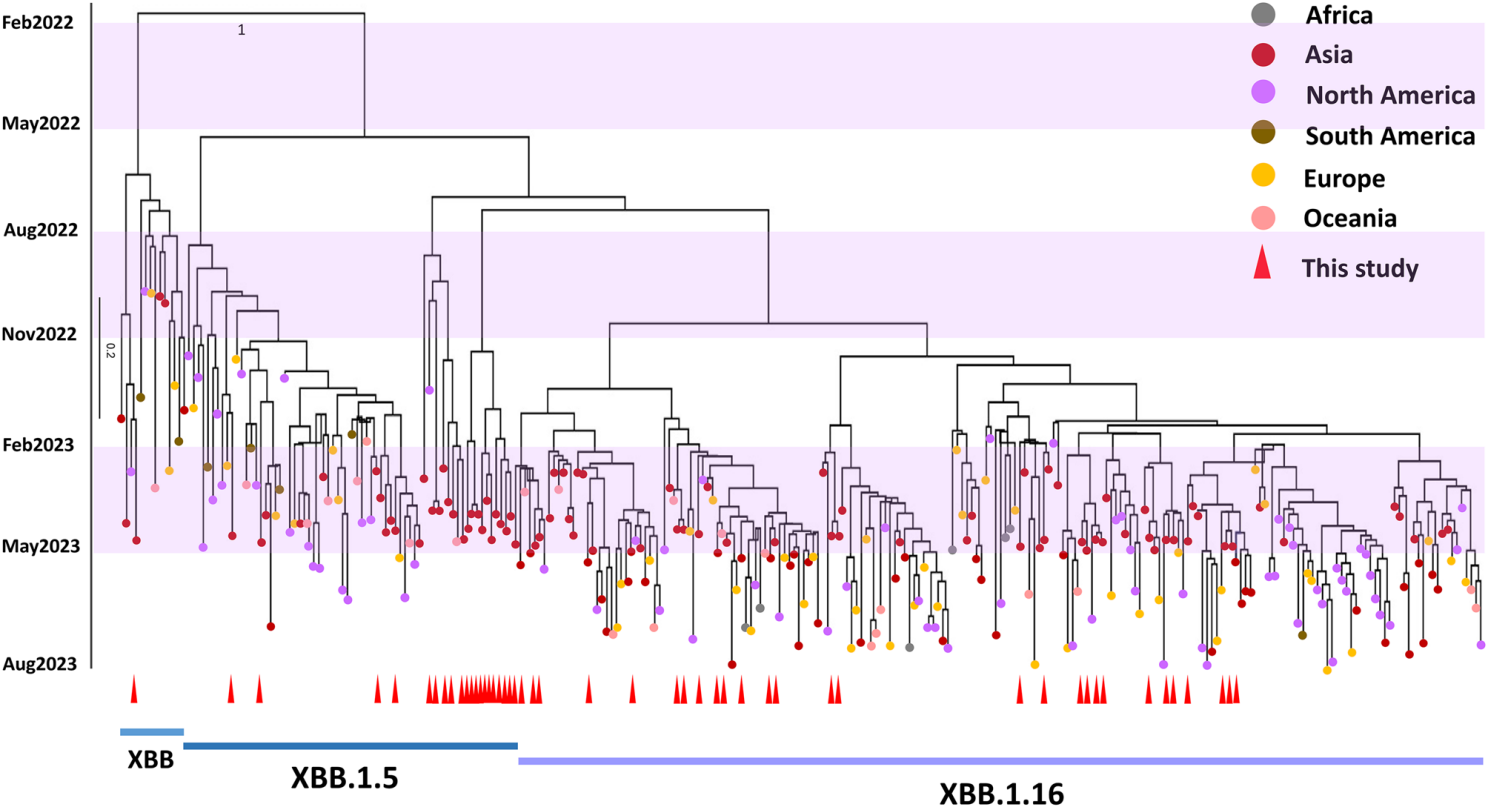
Divergence times of phylogenetic trees of global XBB.1.5, XBB.1.16, and XBB viruses using molecular clock. A time-resolved maximum-likelihood phylogenetic tree of XBB.1.5, XBB.1.16, and XBB samples sequenced worldwide and sequences from this study is shown. The scale bar indicates nucleotide substitutions per site.

Using high-throughput sequencing technologies, an additional set of 50 complete SARS-CoV-2 genomes derived from COVID-19 patients in Thailand was successfully compiled. The time-resolved maximum likelihood tree constructed using global XBB.1.5 and XBB.1.16 sequences in comparison with the parent XBB sequences shown in Fig. 2 indicates that the emergence of the most recent common ancestor of XBB.1.5 can be traced back to approximately June 2022. Based on analysis of the time to the most recent common ancestor, the emergence of XBB.1.16 took place around October 2022. In Fig. 2 the tips are color-annotated based on the continent where the sequences were identified, and the sequences from the current study are labeled with a red triangle. The evolutionary rate was estimated to be 0.73 × 10^−3^ nucleotide substitutions per site per year (sub/site/year) (95% HDI 0.59 × 10^−3^ to 0.85 × 10^−3^) from the XBB.1.5, XBB.1.16, and XBB variant time-scaled phylogeny inferred with the complete genome dataset. The XBB.1.16 variant consisted of 203 sequences and exhibited substantial genetic diversity, forming two distinct subclades in October 2022. This variant also exhibited the broadest geographical distribution worldwide.

**Figure 3:**
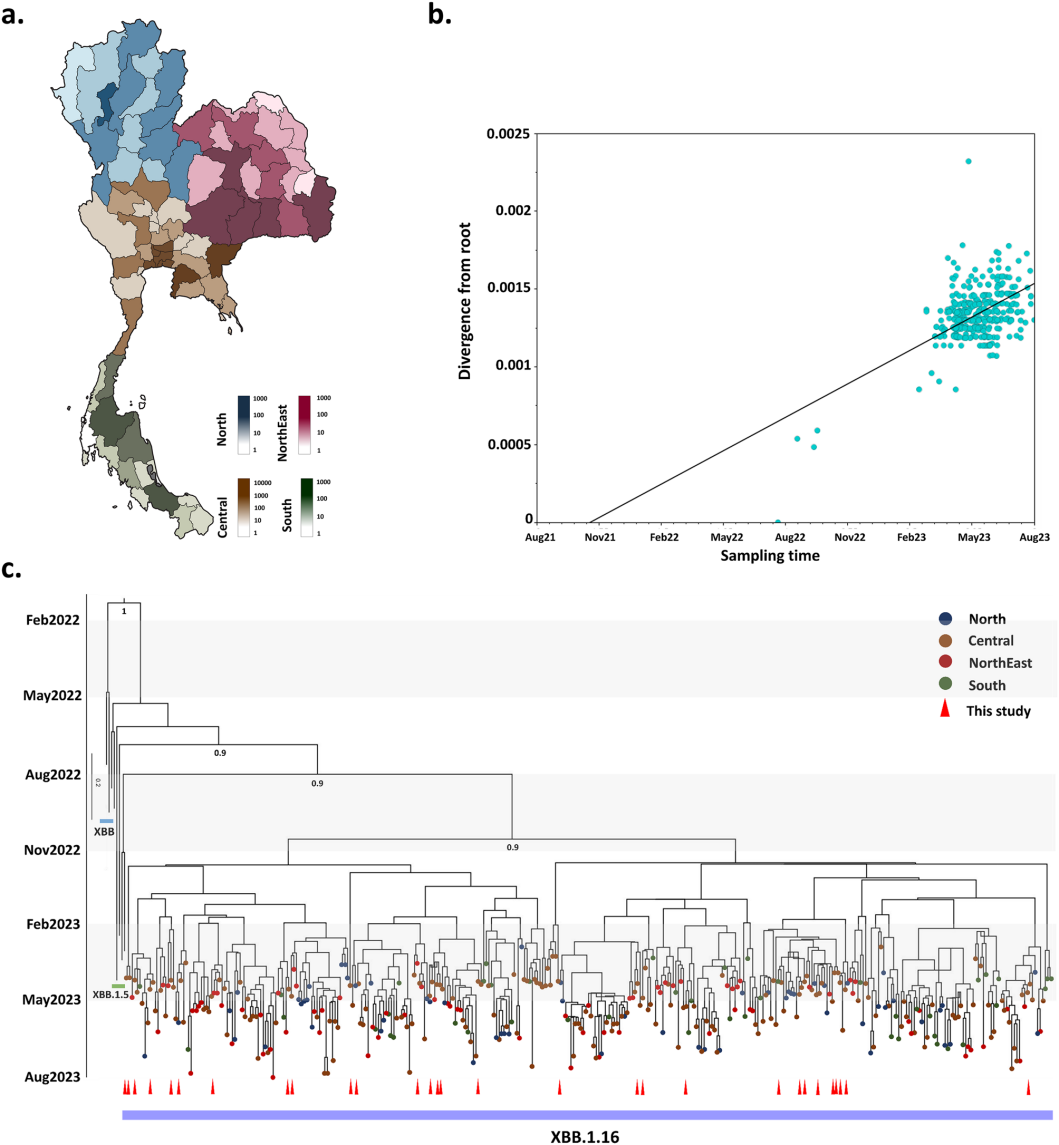
(**a**) Map of the regions in Thailand most affected by the SARS-CoV-2 outbreak from December 2022 to August 2023^31^. Administrative divisions in the northern, central, northeastern, and southern areas are differentiated by blue, brown, red, and green color respectively. These colors represent the cumulative numbers of confirmed and suspected cases reported throughout the outbreak. (**b**) Root-to-tip regression of genetic distances against sampling time (month) for SARS-CoV-2 XBB.1.16 variant sequences based on a maximum likelihood tree. (**c**) Temporal phylogeny of all publicly accessible XBB.1.16 genomes in Thailand estimated using BEAST. Each tip is color-coded based on the probable region within the country where the patient was infected.

SARS-CoV-2 infections have been detected in nearly all provinces across Thailand, except for Phang Nga Province in the southern region, where no cases have been reported (Fig. 3a). The three provinces with the highest numbers of SARS-CoV-2 infections, Bangkok, Chonburi, and Nonthaburi, are all located in the central region. In the northern region, Lamphun had the highest number of infections, and in the southern region, Songkhla and Surat Thani had the highest numbers of infections. In the northeastern region, the provinces with the most infections were Khon Kaen, Nakhon Ratchasima, Buri Ram, Surin, and Ubon Ratchathani (Fig. 3a).

As at 13 August 2023 a total of 329 complete SARS-CoV-2 XBB.1.16 genomes identified in Thailand were accessible via GISAID^32^. Together with the 29 XBB.1.16 sequences acquired from Bangkok Province in the present study, the dataset includes a total of 358 genome sequences of SARS-CoV-2 XBB.1.16 collected from 58 provinces across Thailand, with sampling dates between December 2022 and August 2023. The sequences predominantly originate from Bangkok in the central region (100/358, 27.9%) and Nakhon Phanom in the northeast region (31/358, 8.7%).

Root-to-tip linear regression analyses were conducted using the best-fitting root, which minimizes the mean of the squares of the residuals, to investigate relationships between genetic divergence and sampling dates. These analyses indicated significant temporal structure in the collected dataset (R^2^ = 0.30, correlation coefficient 0.55), suggesting a clocklike pattern of molecular evolution, with a substitution rate of 0.86 × 10^−3^ sub/site/year (Fig. 3b).

Phylogenetic analysis revealed the presence of at least four distinct XBB.1.16 lineages in Thailand (Fig. 3c). The majority of XBB.1.16 genomic sequences sampled from Bangkok Province formed clusters with genomic sequences from several other Thailand provinces. This suggests that XBB.1.16 had already spread from Bangkok Province to other regions in Thailand, indicating multiple independent introductions of XBB.1.16 from various Thailand provinces into Bangkok. Based on Bayesian time-scaled phylogenetic analysis using the tip-dating method, the estimated most recent common ancestor date for XBB.1.16 was January 2022, with an evolutionary rate estimate of 0.79 × 10^−3^ sub/site/year (95% highest posterior density interval 0.46 × 10^−3^ to 1.13 × 10^−3^).

## Discussion

Despite the WHO discontinuing COVID-19’s global emergency status, SARS-CoV-2 continues to cause substantial morbidity and epidemics worldwide, including in Thailand. The current study investigated the molecular epidemiology of SARS-CoV-2 variants and the evolution of XBB.1.16 circulating in Thailand between December 2022 and August 2023. The investigation was conducted by characterizing the weekly occurrences of SARS-CoV-2-associated outbreaks, and analyzing the distribution of circulating variants during these outbreaks. In Thailand during the first half of 2022 the predominant SARS-CoV-2 variants were the omicron subvariants BA.1 and BA.2, with subsequent months seeing an emergence of BA.4 and BA.5, as previously reported^26,33^. The present study revealed that in December 2022 initial identification of the XBB, XBB.1, BA.5.2, and BA.5.3 lineages indicated low prevalence. Between December 2022 and February 2023, BA.2.75 was the most dominant variant among COVID-19 cases, followed by XBB.1 and XBB.1.5. The emergence of XBB.1.5 resulted in its dominance from March to April. By mid-June 2023, XBB.1.16 had ascended as the dominant global strain, surpassing the XBB.1.5 variant in terms of prevalence^28^. As at 05 June 2023 a total of 19,847 sequences belonging to the omicron XBB.1.16 variant were accessible, originating from 66 different countries. A large proportion (40.7%) of these XBB.1.16 sequences were sampled in India. Sequences from other Asian nations are also noteworthy, with contributions from China (6.1%), Singapore (5.6%), Japan (4.5%), South Korea (4.3%), Malaysia (1.5%), Thailand (1.5%), Indonesia (0.7%), Brunei (0.6%), and Vietnam (0.6%)^34^. The present study is consistent with a report from Singapore, which found that during the first half of 2023, XBB.1.16 was the predominant strain, followed by XBB.1.5, XBB.1.9, and XBB.2.3^35^. That report also indicated that there is a higher risk of severe outcomes when infected with XBB.1.16 compared to XBB.1.5 or XBB.1.9, there is no significant difference in the risk of hospitalization associated with different XBB variants.

A previous study investigated the global evolution rate of SARS-CoV-2 during the early stages of the pandemic and reported an estimated mean nucleotide mutation rate of approximately 6.58 × 10^−3^ sub/site/year^36^. The evolution rates for the full genome of the Gamma variant reportedly range from 1.12 × 10^−3^ to 4.91 × 10^−3^ sub/site/year, as determined via sequencing of circulating viruses^37,38^. The evolution rates of XBB.1 and XBB.1.5 variants exhibit a degree of similarity, at 0.63 × 10^−3^ and 0.69 × 10^−3^ sub/site/year respectively^39,40^. In the present study the respective rates of evolutionary change of the XBB.1.16 variant determined via Bayesian analyses with the tip-dating method and root-to-tip linear regression were 0.79 × 10^−3^ sub/site/year and 0.86 × 10^−3^ sub/site/year. Notably, these rates are slightly higher than those reported in another study that investigated XBB.1.16 variants, which documented a rate of 0.39 × 10^−3^ sub/site/year^41^.

In early 2022, Thailand experienced a surge in SARS-CoV-2 infections propelled by the BA.1 and BA.2 variants, resulting in significant outbreaks with weekly cases reaching 150,000–180,000. The following three months witnessed a sharp decline, nearly a tenfold reduction per week, credited to the emergence of the BA.5 variant. However, the recent appearance of the XBB.1.16 variant has led to a substantial increase, with weekly cases surging from 100 to 3000. There is no evidence of a difference in clinical outcomes among individuals infected with different variants in Thailand. This could be challenging to ascertain, considering that a majority of the population has been vaccinated.

The current study had several limitations, with one primary constraint being the potential for sampling bias. The genomic data used in the study were obtained from diagnosed cases, which may not comprehensively represent the entire spectrum of SARS-CoV-2 infections. Asymptomatic patients, mild infections, or cases in regions with limited testing may be underrepresented in the dataset, leading to a skewed understanding of the virus’s evolutionary dynamics. Additionally, because XBB.1.16 is a new variant, limited historical genomic data are available for comparison. While genomic data can reveal mutations and genetic changes, determining their functional implications (*e.g.*, increased transmission, immune escape, and altered disease severity) requires additional experimental studies. Genomic data alone may not completely facilitate understanding of the consequences of specific mutations.

In conclusion, the present study contributes to the understanding of the genomic epidemiology and evolution of XBB.1.16 in Bangkok, Thailand. The dominant sub-lineage that emerged was XBB.1.16, beginning in March 2023. Phylogenetic analysis showed a minimum of four distinct lineages of XBB.1.16, indicating the multiple independent introductions into Bangkok. Our analysis shows the potential of molecular epidemiology and the evolution of XBB.1.16 to identify the most prevalent and potentially emerging strains, which may guide vaccine design to reduce the impact of COVID-19 illness.

## Materials and methods

### Institutional review board statement

The study was conducted in accordance with the guidelines of the Declaration of Helsinki and was approved by the Institutional Review Board of the Faculty of Medicine, Chulalongkorn University, Thailand (approval number IRB0538/66). All information and patient identifiers were anonymized to protect patient confidentiality. Due to the retrospective nature of the study, the Institutional Review Board of the Faculty of Medicine, Chulalongkorn University, Thailand (approval number IRB0538/66) waived the need to obtain informed consent.

### SARS-CoV-2 sample collection and viral RNA extraction

The nasopharyngeal and pharyngeal swabs from 491 Bangkok residents used in this study were submitted to collaborating hospitals for SARS-CoV-2 testing. RNA extraction from a 200-μL aliquot of the supernatant was conducted in our laboratory using a magLEAD 12gC instrument (Precision System Science, Chiba, Japan) in accordance with the manufacturer’s instructions. From December 2022 to August 2023, a total of 491 SARS-CoV-2 RNA samples were collected, and the partial spike gene was amplified from individuals diagnosed with COVID-19. Of those, 50 samples were randomly selected and subjected to complete genome sequencing.

### Partial spike gene amplification and sequencing

A total of 491 samples that tested positive for SARS-CoV-2 were subjected to variant identification via RT-PCR using primers designed based on nucleotide sequences obtained from the GenBank database. The PCR aimed to amplify the partial spike gene with an amplicon length of 1,036 bp, using the following primers:

$$ {\text{spike}}/{\text{F}}22188;\;5^{\prime} - {\text{CTACTGATGCTGTCCGTGATCCAC}} - 3^{\prime} $$

$$ {\text{spike}}/{\text{R}}23223;\;5^{\prime} - {\text{TCAGTAAGAACACCTGTGCCTT}} - 3^{\prime} $$

Briefly, RT-PCR was conducted in a total volume of 25 µL, incorporating 2–3 µL of 100 ng to 1 µg of total RNA, 0.5 µM of each primer, 12.5 µL of 2X Reaction Mix (containing 0.4 mM of each dNTP and 3.2 mM MgSO_4_), 1 µL of SSIII RT/Platinum Taq Mix, and nuclease-free water. The Superscript III One-Step RT-PCR system with Platinum Taq High Fidelity was used in accordance with the manufacturer’s instructions (Invitrogen, Carlsbad, CA, USA). The PCR process included initial incubation at 45 °C for 40 min, followed by 40 cycles consisting of denaturation at 94 °C for 30 s, annealing at 50 °C for 30 s, and extension at 68 °C for 1 min 45 s. A final extension was performed at 68 °C for 5 min. Both forward and reverse directions were employed simultaneously for sequencing and product amplification at First BASE Laboratories Sdn Bhd (Selangor Darul Ehsan, Malaysia).

#### NGS

The Celemics comprehensive respiratory virus panel (Celemics Inc., Incheon, Republic of Korea) was utilized for sequencing and identifying complete SARS-CoV-2 genomes. In the RNA extraction process, 25 ng of extracted RNA was mixed with an RNA fragment buffer mix to facilitate fragmentation. Subsequently, first-strand cDNA was synthesized using a first-strand synthesis master mix. The first-strand cDNA underwent double-stranded cDNA construction through incubation at 16 °C for 60 min with a 2nd-strand synthesis-1 mix, followed by a 2nd-strand synthesis-2 mix at 25 °C for 15 min. The resulting double-stranded cDNA underwent cleaning, repair, and the addition of poly(A) tail oligomers in a 5 ERA buffer mix. Following multiple incubation steps at different temperatures, the A-tailed DNA was ligated with adaptors in a ligation reaction mix at 20 °C for 15 min. The ligated DNA was purified using CeleMag cleanup beads, amplified, and transformed into an adaptor-ligated library using CLM polymerase and UDI primers, following the manufacturer’s instructions. To assess the quantity and quality of the constructed DNA library, automated capillary gel electrophoresis (QIAxcel; Qiagen, Hilden, Germany) was employed, ensuring the presence of 200- to 400-bp DNA fragments. The DNA libraries were subsequently subjected to Next-Generation Sequencing (NGS) using the Illumina NextSeq 500 system with the mid/high-output kit v2.5 (300 cycles). The resulting FASTQ data underwent trimming, assembly, and analysis through the Celemics Virus Verifier pipeline, facilitating identifying and generating consensus sequences for the SARS-CoV-2 genome. Any nucleotide gaps identified in the assembled SARS-CoV-2 FASTQ sequences were addressed by incorporating nucleotide sequences obtained from conventional RT-PCR-derived Sanger sequencing. This was accomplished using primers specifically designed for those gaps.

### Bayesian phylogenetic analysis

By 13 August 2023, a set of high-quality SARS-CoV-2 genomes had been retrieved from GISAID. These genomes met specific criteria, including having < 1% unidentified nucleotides (N) and a complete length > 29 kb. The sampled genomes represented the lineages XBB (*n* = 13), XBB.1.5 (*n* = 44), and XBB.1.16 (*n* = 188) globally, and there were 329 samples of lineage XBB.1.16 from Thailand from the respective lineages covering the same timeframe (December 2022–August 2023). SARS-CoV-2 XBB.1.5 and XBB.1.16 complete genome sequences from Thailand were compiled using BioEdit v.7.2.6 software^42^ and aligned with the global reference datasets of the corresponding lineage using MAFFT v.7.475^43^.

BEAST v.1.10.5^44^ was employed for constructing Bayesian time-scaled phylogenies. These phylogenies utilized a general time-reversible substitution model with gamma-distributed rate variation among sites, a relaxed molecular clock with a log-normal prior^45^, and an exponential growth coalescent tree prior^46^. Two independent Markov Chain Monte Carlo (MCMC) chains, each comprising 200 million states, were merged using the BEAGLE package^47^ to enhance computational efficiency and runtime. Sampling of parameters and trees occurred every 20,000 steps, discarding the initial 20% as burn-in. To ensure an effective sample size > 200 for all estimated parameters, convergence and mixing of MCMC chains were assessed using Tracer v.1.7^48^. Maximum clade credibility (MCC) trees were summarized using TreeAnnotator v.1.10, and visualized with FigTree v.1.4.4 (https://github.com/rambaut/figtree/releases).

### Maximum likelihood phylogenetic tree and temporal signal

The XBB.1.16 dataset underwent maximum likelihood phylogenetic analysis with IQ TREE v.2.1.2^49^. This analysis utilized a general time-reversible model of nucleotide substitution with gamma-distributed rate variation among sites (G4). It included a proportion of invariable sites (+ I) and empirical base frequencies (+ F), chosen through recommendations from ModelFinder within IQ-TREE^50^. Branch support was assessed using the Shimodaira-Hasegawa-like procedure (SH-aLRT) with approximately 1000 replicates for the likelihood-ratio test. To assess the temporal signal within the XBB.1.16 dataset, regression analysis of root-to-tip divergence against sampling time was performed using TempEst v.1.5.36^51^, based on the ML tree.

### Nucleotide sequence accession IDs

The genome sequences obtained in this study have been submitted to the NCBI databases. Accession numbers are provided in Supplementary Table S1.

### Supplementary Information

Supplementary Information.

## Data Availability

The authors confirm that the data supporting the findings of this study are available within the article.
