# Supplementary material for: Genomic epidemiology and evolutionary analysis during XBB.1.16-predominant periods of SARS-CoV-2 omicron variant in Bangkok, Thailand: December 2022–August 2023
Source: Sci Rep. 2024 Jan 5;14:645. doi: 10.1038/s41598-023-50856-0 (PMC10770311; doi:10.1038/s41598-023-50856-0)
Supplement: Supplementary file 1 — Supplementary Information. [file 41598_2023_50856_MOESM1_ESM.pdf]

Supplement Figure 1. Timeline showing the SARS-CoV-2 variant in Thailand, 2021-2023 [16-19].

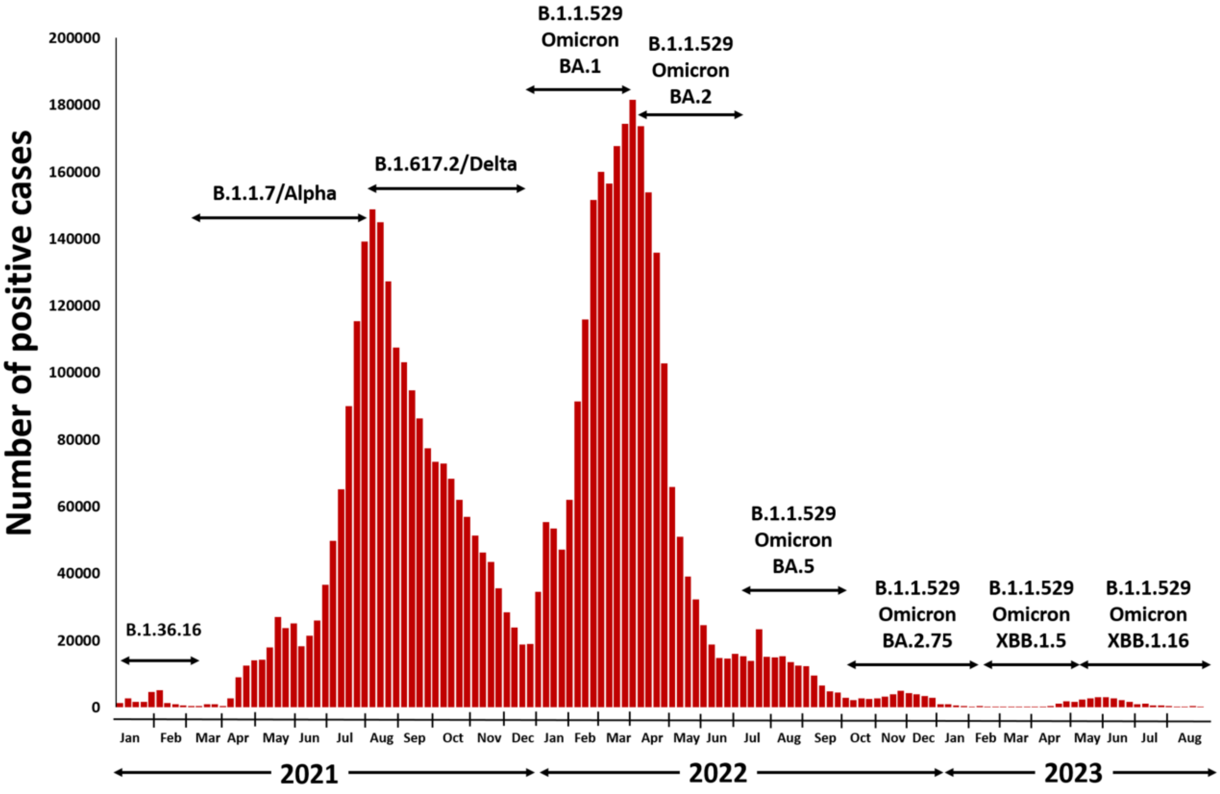

Supplement Figure 2. Divergence times of phylogenetic trees of 491 Thai SARS-CoV-2 partial spike sequences using molecular clock. The scale bar indicates nucleotide substitutions per site.

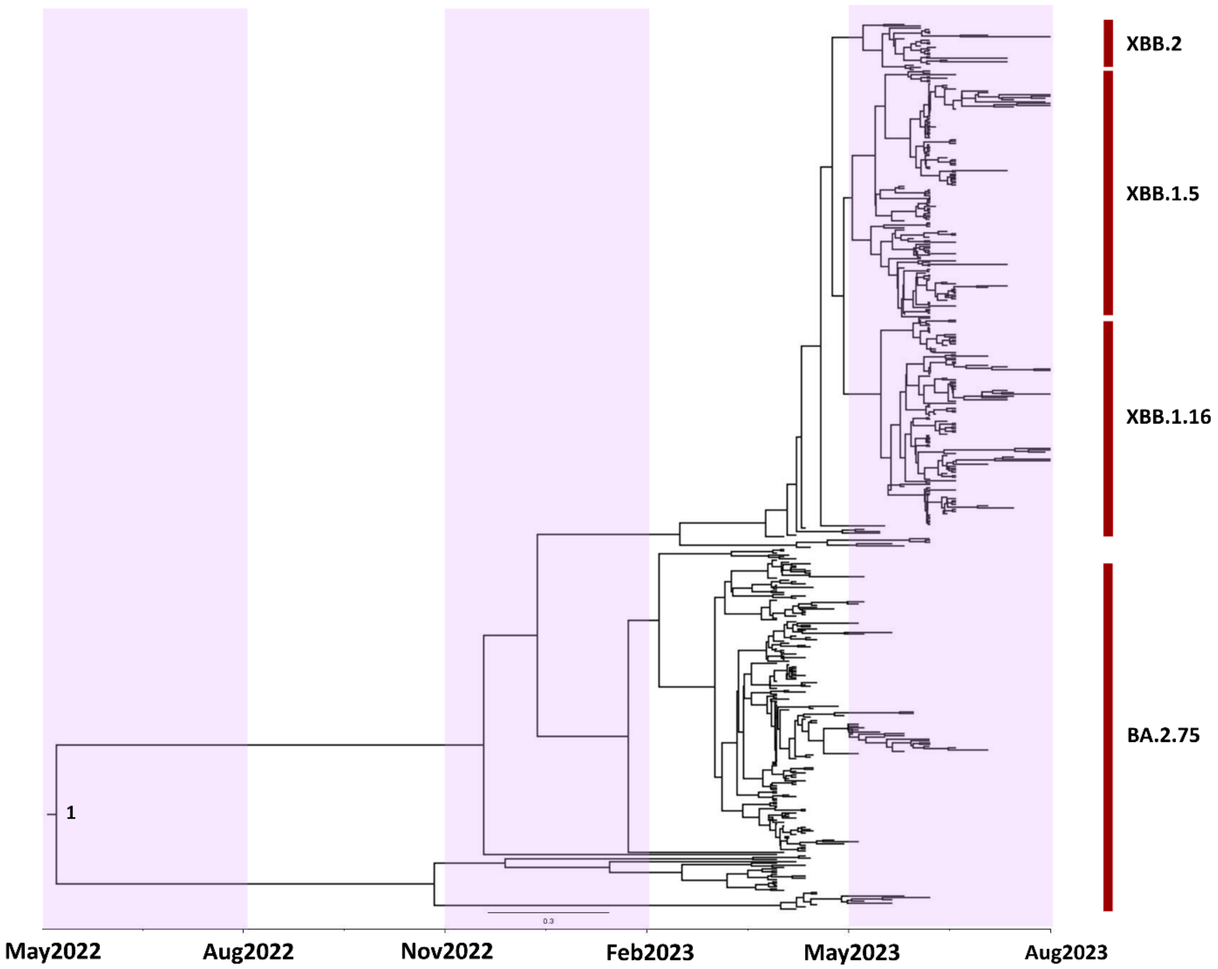

Supplement Table 1. NCBI Accession Numbers.

No	Isolate	Accession	Collection date
1	hCoV-19/Thailand/Bangkok_CU10430/2023	OR608405	27-Feb-23
2	hCoV-19/Thailand/Bangkok_CU10438/2023	OR608406	15-Mar-23
3	hCoV-19/Thailand/Bangkok_CU10441/2023	OR608407	26-Mar-23
4	hCoV-19/Thailand/Bangkok_CU10442/2023	OR608408	26-Mar-23
5	hCoV-19/Thailand/Bangkok_CU10444/2023	OR608409	18-Mar-23
6	hCoV-19/Thailand/Bangkok_CU10447/2023	OR608410	24-Mar-23
7	hCoV-19/Thailand/Bangkok_CU10448/2023	OR608411	24-Mar-23
8	hCoV-19/Thailand/Bangkok_CU10449/2023	OR608412	28-Mar-23
9	hCoV-19/Thailand/Bangkok_CU10451/2023	OR608413	27-Mar-23
10	hCoV-19/Thailand/Bangkok_CU10456/2023	OR608414	13-Mar-23
11	hCoV-19/Thailand/Bangkok_CU10462/2023	OR608415	29-Mar-23
12	hCoV-19/Thailand/Bangkok_CU10463/2023	OR608416	10-Apr-23
13	hCoV-19/Thailand/Bangkok_CU10465/2023	OR608417	10-Apr-23
14	hCoV-19/Thailand/Bangkok_CU10468/2023	OR608418	10-Apr-23
15	hCoV-19/Thailand/Bangkok_CU10469/2023	OR608419	10-Apr-23
16	hCoV-19/Thailand/Bangkok_CU10470/2023	OR608420	15-Apr-23
17	hCoV-19/Thailand/Bangkok_CU10471/2023	OR608421	18-Apr-23
18	hCoV-19/Thailand/Bangkok_CU10472/2023	OR608422	18-Apr-23
19	hCoV-19/Thailand/Bangkok_CU10473/2023	OR608423	18-Apr-23
20	hCoV-19/Thailand/Bangkok_CU10480/2023	OR608424	18-Apr-23
21	hCoV-19/Thailand/Bangkok_CU10486/2023	OR608425	18-Apr-23
22	hCoV-19/Thailand/Bangkok_CU10492/2023	OR608426	08-Apr-23
23	hCoV-19/Thailand/Bangkok_CU10493/2023	OR608427	08-Apr-23
24	hCoV-19/Thailand/Bangkok_CU10495/2023	OR608428	08-Apr-23
25	hCoV-19/Thailand/Bangkok_CU10500/2023	OR608429	12-Apr-23
26	hCoV-19/Thailand/Bangkok_CU10510/2023	OR608430	21-Apr-23
27	hCoV-19/Thailand/Bangkok_CU10512/2023	OR608431	21-Apr-23
28	hCoV-19/Thailand/Bangkok_CU10516/2023	OR608432	22-Apr-23
29	hCoV-19/Thailand/Bangkok_CU10525/2023	OR608433	21-Apr-23
30	hCoV-19/Thailand/Bangkok_CU10531/2023	OR608434	19-Apr-23
31	hCoV-19/Thailand/Bangkok_CU10534/2023	OR608435	19-Apr-23
32	hCoV-19/Thailand/Bangkok_CU10535/2023	OR608436	24-Apr-23
33	hCoV-19/Thailand/Bangkok_CU10541/2023	OR608437	15-Apr-23
34	hCoV-19/Thailand/Bangkok_CU10542/2023	OR608438	15-Apr-23
35	hCoV-19/Thailand/Bangkok_CU10551/2023	OR608439	20-Apr-23
36	hCoV-19/Thailand/Bangkok_CU10554/2023	OR608440	20-Apr-23
37	hCoV-19/Thailand/Bangkok_CU10555/2023	OR608441	20-Apr-23
38	hCoV-19/Thailand/Bangkok_CU10560/2023	OR608442	20-Apr-23
39	hCoV-19/Thailand/Bangkok_CU10561/2023	OR608443	20-Apr-23

40	hCoV-19/Thailand/Bangkok_CU10567/2023	OR608444	23-Apr-23
41	hCoV-19/Thailand/Bangkok_CU10570/2023	OR608445	23-Apr-23
42	hCoV-19/Thailand/Bangkok_CU10571/2023	OR608446	23-Apr-23
43	hCoV-19/Thailand/Bangkok_CU10580/2023	OR608447	29-Apr-23
44	hCoV-19/Thailand/Bangkok_CU10591/2023	OR608448	28-Apr-23
45	hCoV-19/Thailand/Bangkok_CU10593/2023	OR608449	28-Apr-23
46	hCoV-19/Thailand/Bangkok_CU10626/2023	OR608450	03-May-23
47	hCoV-19/Thailand/Bangkok_CU10630/2023	OR608451	03-May-23
48	hCoV-19/Thailand/Bangkok_CU10635/2023	OR608452	08-May-23
49	hCoV-19/Thailand/Bangkok_CU10636/2023	OR608453	06-May-23
50	hCoV-19/Thailand/Bangkok_CU10638/2023	OR608454	06-May-23

Supplement Table 2. The GISAID IDs for 329 complete SARS-CoV-2 XBB.1.16 genomes identified in Thailand

No.	GISAID ID	Province	Region	Collection Date
1	17359413	Bangkok	Central	20-03-23
2	17413017	Bangkok	Central	20-03-23
3	17513923	Bangkok	Central	31-03-23
4	17513924	Songkhla	South	21-03-23
5	17513932	Bangkok	Central	22-03-23
6	17513935	Bangkok	Central	27-03-23
7	17596756	Krabi	South	28-03-23
8	17640455	Nakhon Phanom	NortEast	28-03-23
9	17994655	Bangkok	Central	07-03-23
10	17367345	Chiang Rai	North	07-03-23
11	17517561	Chiang Rai	North	29-03-23
12	17517563	Chiang Rai	North	29-03-23
13	17417436	Trang	South	22-03-23
14	17513925	Chon Buri	East	02-04-23
15	17513927	Chon Buri	East	03-04-23
16	17547359	Ang Thong	Central	10-04-23
17	17596537	Chon Buri	East	11-04-23
18	17596708	Trang	South	16-04-23
19	17596712	Krabi	South	17-04-23
20	17596728	Bangkok	Central	18-04-23
21	17596751	Bangkok	Central	17-04-23
22	17602912	Songkhla	South	21-04-23
23	17602923	SamutSongkhram	Central	22-04-23
24	17602933	NakhonSawan	Central	17-04-23
25	17602938	Singburi	Central	21-04-23
26	17602942	NakhonRatchasima	NortEast	24-04-23
27	17602946	Bangkok	Central	25-04-23
28	17602948	UbonRatchathani	NortEast	25-04-23
29	17602957	Tak	West	25-04-23
30	17602976	MahaSarakham	NortEast	19-04-23
31	17602977	Maha Sarakham	NortEast	19-04-23
32	17622123	Nonthaburi	Central	27-04-23
33	17622137	Bangkok	Central	30-04-23
34	17622152	ChiangMai	North	25-04-23
35	17622160	Lampang	North	21-04-23
36	17622174	SuratThani	South	23-04-23
37	17622176	NakhonSiThammarat	South	19-04-23
38	17622179	Chumphon	South	20-04-23

39	17622208	Satun	South	25-04-23
40	17622218	Uttaradit	North	21-04-23
41	17622228	LopBuri	Central	22-04-23
42	17622229	Saraburi	Central	26-04-23
43	17622233	SamutPrakan	Central	28-04-23
44	17622238	KhonKaen	NortEast	23-04-23
45	17622246	NongKhai	NortEast	26-04-23
46	17622250	SakonNakhon	NortEast	26-04-23
47	17622263	Bangkok	Central	21-04-23
48	17623935	KhonKaen	NortEast	26-04-23
49	17623943	SuratThani	South	19-04-23
50	17623947	SuratThani	South	10-04-23
51	17624061	Bangkok	Central	17-04-23
52	17624075	Bangkok	Central	20-04-23
53	17624086	Bangkok	Central	22-04-23
54	17640474	NakhonPhanom	NortEast	18-04-23
55	17640483	Nakhon Phanom	NortEast	19-04-23
56	17640502	Nakhon Phanom	NortEast	20-04-23
57	17661686	SamutSakhon	Central	24-04-23
58	17661735	KhonKaen	NortEast	28-04-23
59	17734697	SuratThani	South	02-04-23
60	17791607	NakhonPhanom	NortEast	25-04-23
61	17791641	Nakhon Phanom	NortEast	25-04-23
62	17959881	Bangkok	Central	12-04-23
63	17994650	ChiangMai	North	07-04-23
64	17554918	Nonthaburi	Central	20-04-23
65	17596729	SuratThani	South	19-04-23
66	17602921	ChiangMai	North	21-04-23
67	17602936	AngThong	Central	18-04-23
68	17602971	ChiangMai	North	21-04-23
69	17603088	ChiangMai	North	08-04-23
70	17622122	Nonthaburi	Central	29-04-23
71	17622159	ChiangMai	North	24-04-23
72	17622219	KamphaengPhet	Central	27-04-23
73	17622244	Loei	NortEast	26-04-23
74	17837198	Tak	West	21-04-23
75	17602956	Tak	West	25-04-23
76	17602966	ChiangRai	North	15-04-23
77	17602979	MahaSarakham	NortEast	04-04-23
78	17596713	Krabi	South	17-04-23
79	17596711	Krabi	South	17-04-23

80	17692248	Bangkok	Central	01-05-23
81	17692253	Bangkok	Central	01-05-23
82	17709517	Ayutthaya	Central	01-05-23
83	17734605	Ayutthaya	Central	08-05-23
84	17734611	Bangkok	Central	09-05-23
85	17734614	Bangkok	Central	12-05-23
86	17734628	Kalasin	NortEast	18-05-23
87	17734629	Lampang	North	12-05-23
88	17734631	MahaSarakham	NortEast	24-05-23
89	17734632	MahaSarakham	NortEast	23-05-23
90	17734634	Nonthaburi	Central	21-05-23
91	17734635	Nakhon Ratchasima	NortEast	08-05-23
92	17734637	NakhonPhanom	NortEast	02-05-23
93	17734645	RoiEt	NortEast	19-05-23
94	17734646	Songkhla	South	01-05-23
95	17734681	Mukdahan	NortEast	23-05-23
96	17734692	Phayao	North	15-05-23
97	17734693	SuratThani	South	12-05-23
98	17734699	SamutPrakan	Central	21-05-23
99	17735480	UdonThani	NortEast	23-05-23
100	17735481	Yasothon	NortEast	15-05-23
101	17774450	Phitsanulok	North	17-05-23
102	17774453	Uttaradit	North	24-05-23
103	17774455	SamutPrakan	Central	25-05-23
104	17774471	KamphaengPhet	Central	18-05-23
105	17774478	KamphaengPhet	Central	17-05-23
106	17774484	UthaiThani	Central	24-05-23
107	17774508	UbonRatchathani	NortEast	19-05-23
108	17785868	Ayutthaya	Central	18-05-23
109	17785870	NakhonPhanom	NortEast	24-05-23
110	17785875	Chanthaburi	East	15-05-23
111	17785879	SuratThani	South	18-05-23
112	17785880	Bangkok	Central	18-05-23
113	17785886	SamutSakhon	Central	15-05-23
114	17785899	Krabi	South	22-05-23
115	17785906	Lampang	North	19-05-23
116	17785911	Trang	South	31-05-23
117	17785915	Bangkok	Central	30-05-23
118	17785920	Bangkok	Central	18-05-23
119	17785923	Bangkok	Central	11-05-23
120	17785927	Bangkok	Central	19-05-23

121	17791617	NakhonPhanom	NortEast	02-05-23
122	17791619	Nakhon Phanom	NortEast	02-05-23
123	17791621	Nakhon Phanom	NortEast	02-05-23
124	17791625	Nakhon Phanom	NortEast	03-05-23
125	17791631	NakhonPhanom	NortEast	09-05-23
126	17811403	Bangkok	Central	04-05-23
127	17811418	Bangkok	Central	09-05-23
128	17811430	Bangkok	Central	11-05-23
129	17816146	SamutSongkhram	Central	30-05-23
130	17816152	Sukhothai	Central	31-05-23
131	17816166	Nan	North	24-05-23
132	17816171	Chon Buri	East	06-06-23
133	17816277	Lop Buri	Central	30-05-23
134	17816290	Bangkok	Central	23-05-23
135	17816300	NakhonRatchasima	NortEast	29-05-23
136	17816315	UthaiThani	Central	29-05-23
137	17816354	Krabi	South	28-05-23
138	17832523	NakhonPhanom	NortEast	08-05-23
139	17832546	NakhonPhanom	NortEast	10-05-23
140	17832561	NakhonPhanom	NortEast	15-05-23
141	17832582	NakhonPhanom	NortEast	19-05-23
142	17837221	Tak	North	02-05-23
143	17837231	NakhonPhanom	NortEast	19-05-23
144	17851230	SuphanBuri	Central	31-05-23
145	17851250	Lampang	North	31-05-23
146	17851395	Songkhla	South	31-05-23
147	17959848	Bangkok	Central	15-05-23
148	17959857	Bangkok	Central	19-05-23
149	17959869	Bangkok	Central	22-05-23
150	17959871	Bangkok	Central	23-05-23
151	17959876	Bangkok	Central	24-05-23
152	17981717	Tak	North	09-05-23
153	17981740	NakhonPhanom	NortEast	22-05-23
154	17981754	NakhonPhanom	NortEast	24-05-23
155	17981755	NakhonPhanom	NortEast	24-05-23
156	17981768	NakhonPhanom	NortEast	29-05-23
157	17981776	NakhonPhanom	NortEast	31-05-23
158	17994638	Bangkok	Central	30-05-23
159	18007781	Bangkok	Central	25-05-23
160	18007789	Bangkok	Central	27-05-23
161	18007796	Bangkok	Central	31-05-23

162	18106601	Bangkok	Central	06-05-23
163	18106602	Bangkok	Central	10-05-23
164	17661725	Bangkok	Central	04-05-23
165	17734655	Satun	South	08-05-23
166	17774457	NongKhai	NortEast	27-05-23
167	17785872	ChiangRai	North	11-05-23
168	17785892	PrachuapKhiriKhan	South	31-05-23
169	17785905	Samut Prakan	Central	19-05-23
170	17785907	Lampang	North	21-05-23
171	17791623	NakhonPhanom	NortEast	03-05-23
172	17811402	Bangkok	Central	04-05-23
173	17811431	Bangkok	Central	12-05-23
174	17816151	Phitsanulok	North	27-05-23
175	17819901	SamutSakhon	Central	25-05-23
176	17837222	Tak	North	02-05-23
177	17837218	Tak	North	01-05-23
178	17837223	Tak	North	02-05-23
179	17837224	Tak	North	03-05-23
180	17981713	Tak	North	08-05-23
181	17661733	Songkhla	South	04-05-23
182	17709556	Nonthaburi	Central	12-05-23
183	17734615	Buriram	NortEast	08-05-23
184	17959851	Bangkok	Central	16-05-23
185	17981749	NakhonPhanom	NortEast	24-05-23
186	17734684	Nong Khai	NortEast	23-05-23
187	17981758	NakhonPhanom	NortEast	25-05-23
188	17981750	NakhonPhanom	NortEast	24-05-23
189	17981737	Tak	North	15-05-23
190	17981736	Tak	North	12-05-23
191	17981730	Tak	North	11-05-23
192	17981720	Tak	North	10-05-23
193	17785936	Bangkok	Central	03-06-23
194	17785943	Bangkok	Central	01-06-23
195	17785956	Phitsanulok	North	06-06-23
196	17785970	ChonBuri	East	01-06-23
197	17816139	SamutSongkhram	Central	01-06-23
198	17816171	Chon Buri	East	06-06-23
199	17816187	ChiangMai	North	08-06-23
200	17816188	Chon Buri	East	07-06-23
201	17816278	Ang Thong	Central	05-06-23
202	17816297	Ayutthaya	Central	08-06-23

203	17816310	UthaiThani	Central	02-06-23
204	17816317	NakhonSawan	Central	02-06-23
205	17816349	Uttaradit	North	05-06-23
206	17816353	Krabi	South	03-06-23
207	17816355	Krabi	South	07-06-23
208	17816359	Chumphon	South	01-06-23
209	17851235	PrachuapKhiriKhan	South	04-06-23
210	17851237	SamutSongkhram	Central	07-06-23
211	17851256	Chon Buri	East	09-06-23
212	17851261	Chon Buri	East	12-06-23
213	17851265	PathumThani	Central	19-06-23
214	17851269	KamphaengPhet	Central	09-06-23
215	17851271	KamphaengPhet	Central	09-06-23
216	17851272	KamphaengPhet	Central	09-06-23
217	17980178	UbonRatchathani	NortEast	26-06-23
218	17980177	UbonRatchathani	NortEast	20-06-23
219	17980169	Chanthaburi	East	12-06-23
220	17980152	ChiangMai	North	19-06-23
221	18007818	Bangkok	Central	05-06-23
222	17953378	Bangkok	Central	14-06-23
223	17953279	BuengKan	NortEast	27-06-23
224	17953271	Prachinburi	East	21-06-23
225	17953251	ChiangRai	North	14-06-23
226	17851293	SakonNakhon	NortEast	20-06-23
227	17851259	Chanthaburi	East	06-06-23
228	17851254	Trat	East	01-06-23
229	17851243	Pathum Thani	Central	15-06-23
230	17816341	NongBuaLamphu	NortEast	13-06-23
231	17816275	Songkhla	South	01-06-23
232	18106598	Bangkok	Central	17-06-23
233	18106586	Bangkok	Central	10-06-23
234	18007819	Bangkok	Central	06-06-23
235	18007814	Bangkok	Central	03-06-23
236	18007807	Bangkok	Central	01-06-23
237	17994668	Krabi	South	25-06-23
238	17994646	Satun	South	28-06-23
239	18106599	Bangkok	Central	17-06-23
240	18106595	Bangkok	Central	14-06-23
241	18106591	Bangkok	Central	12-06-23
242	18106588	Bangkok	Central	10-06-23
243	18106583	Bangkok	Central	09-06-23

244	18106581	Bangkok	Central	09-06-23
245	18106576	Bangkok	Central	07-06-23
246	18042012	Saraburi	Central	28-06-23
247	18007815	Bangkok	Central	04-06-23
248	18007803	Bangkok	Central	01-06-23
249	17994690	NakhonPhanom	NortEast	22-06-23
250	17994687	Chiang Rai	North	21-06-23
251	17994670	Krabi	South	25-06-23
252	17994667	NakhonPhanom	NortEast	19-06-23
253	17994663	KhonKaen	NortEast	21-06-23
254	17994634	PathumThani	Central	19-06-23
255	17980197	Bangkok	Central	23-06-23
256	17980190	Krabi	South	16-06-23
257	17980179	NakhonSiThammarat	South	08-06-23
258	17980171	Rayong	East	26-06-23
259	17980168	UthaiThani	Central	26-06-23
260	17980165	KamphaengPhet	Central	29-06-23
261	17980160	Songkhla	South	26-06-23
262	17980159	Songkhla	South	24-06-23
263	17980158	Uttaradit	North	22-06-23
264	17980157	Uttaradit	North	22-06-23
265	17980156	Phitsanulok	North	22-06-23
266	17980155	Phitsanulok	North	27-06-23
267	17980145	SamutSongkhram	Central	20-06-23
268	17953287	Sukhothai	Central	12-06-23
269	17953252	ChiangRai	North	09-06-23
270	17851393	Singburi	Central	08-06-23
271	17851310	Phitsanulok	North	20-06-23
272	17851306	Kalasin	NortEast	20-06-23
273	17851305	KhonKaen	NortEast	20-06-23
274	17851303	SuratThani	South	20-06-23
275	17851298	Krabi	South	20-06-23
276	17851292	NongBuaLamphu	NortEast	20-06-23
277	17851280	Bangkok	Central	13-06-23
278	17851274	KamphaengPhet	Central	09-06-23
279	18112653	Bangkok	Central	08-08-23
280	18112652	Bangkok	Central	08-08-23
281	18112614	Bangkok	Central	24-07-23
282	18042015	MaeHongSon	North	03-07-23
283	18042025	Nonthaburi	Central	14-07-23
284	18042027	Songkhla	South	04-07-23

285	18042028	Songkhla	South	09-07-23
286	18042039	Sukhothai	Central	07-07-23
287	18042060	Krabi	South	03-07-23
288	18042065	Mukdahan	NortEast	06-07-23
289	18042066	Mukdahan	NortEast	06-07-23
290	18064504	SamutSongkhram	Central	13-07-23
291	18064638	Sukhothai	Central	12-07-23
292	18064547	LopBuri	Central	05-07-23
293	18064546	Phrae	North	16-07-23
294	18064540	Mukdahan	NortEast	20-07-23
295	18064535	KhonKaen	NortEast	18-07-23
296	18064529	Uttaradit	North	14-07-23
297	18064515	Bangkok	Central	19-07-23
298	18064514	Bangkok	Central	21-07-23
299	18064512	Lampang	North	11-07-23
300	18064511	Lampang	North	06-07-23
301	18064506	SamutSongkhram	Central	08-07-23
302	18064503	Phetchaburi	South	10-07-23
303	18064498	KamphaengPhet	Central	01-07-23
304	18042076	Phayao	North	05-07-23
305	18042069	NongBuaLamphu	NortEast	18-07-23
306	18042067	Mukdahan	NortEast	10-07-23
307	18042066	Mukdahan	NortEast	10-07-23
308	18112601	NakhonPhanom	NortEast	03-07-23
309	18112603	NakhonPhanom	NortEast	07-07-23
310	18112604	ChiangRai	North	07-07-23
311	18112605	ChiangRai	North	10-07-23
312	18112617	Lampang	North	18-07-23
313	18112621	Bangkok	Central	07-08-23
314	18112623	ChonBuri	East	31-07-23
315	18112626	LopBuri	Central	21-07-23
316	18112629	NakhonNayok	Central	19-07-23
317	18112633	Saraburi	Central	24-07-23
318	18112634	Kalasin	NortEast	02-08-23
319	18112637	Mukdahan	NortEast	08-08-23
320	18112639	Phitsanulok	North	26-07-23
321	18112642	SakonNakhon	NortEast	24-07-23
322	18112678	SamutSongkhram	Central	03-08-23
323	18115491	Bangkok	Central	07-07-23
324	17661758	Chiang Rai	North	01-05-23
325	18112600	Chiang Rai	North	29-06-23

326	18106587	Bangkok	Central	10-06-23
327	17980139	Ang Thong	Central	21-06-23
328	17816323	AmnatCharoen	NortEast	06-06-23
329	17709807	NakhonSiThammarat	South	28-04-23
